# Incorporating Graphene Nanoplatelets and Carbon Nanotubes in Biobased Poly(ethylene 2,5-furandicarboxylate): Fillers’ Effect on the Matrix’s Structure and Lifetime

**DOI:** 10.3390/polym15020401

**Published:** 2023-01-12

**Authors:** Dimitra Kourtidou, Dimitrios Karfaridis, Thomas Kehagias, George Vourlias, Dimitrios N. Bikiaris, Konstantinos Chrissafis

**Affiliations:** 1 School of Physics, Aristotle University of Thessaloniki, GR-541 24 Thessaloniki, Greece; 2 Laboratory of Polymer Chemistry and Technology, Department of Chemistry, Aristotle University of Thessaloniki, GR-541 24 Thessaloniki, Greece

**Keywords:** polymer nanocomposites, poly(ethylene 2,5-furandicarboxylate) (PEF), carbon nanofillers, structural analysis, lifetime predictions

## Abstract

Poly(ethylene 2,5-furandicarboxylate) (PEF) nanocomposites reinforced with Graphene nanoplatelets (GNPs) and Carbon nanotubes (CNTs) were in situ synthesized in this work. PEF is a biobased polyester with physical properties and is the sustainable counterpart of Polyethylene Terephthalate (PET). Its low crystallizability affects the processing of the material, limiting its use to packaging, films, and textile applications. The crystallization promotion and the reinforcement of PEF can lead to broadening its potential applications. Therefore, PEF nanocomposites reinforced with various loadings of GNPs, CNTs, and hybrids containing both fillers were prepared, and the effect of each filler on their structural characteristics was investigated by X-ray Diffraction (XRD), Fourier transform infrared spectroscopy—attenuated total reflectance (FTIR–ATR), and X-Ray Photoelectron Spectroscopy (XPS). The morphology and structural properties of a hybrid PEF nanocomposite were evaluated by Transmission Electron Microscopy (TEM). The thermo-oxidative degradation, as well as lifetime predictions of PEF nanocomposites, in an ambient atmosphere, were studied using Thermogravimetric Analysis (TGA). Results showed that the fillers’ incorporation in the PEF matrix induced changes in the lamellar thickness and increased crystallinity up to 27%. TEM analysis indicated the formation of large CNTs aggregates in the case of the hybrid PEF nanocomposite as a result of the ultrasonication process. Finally, the presence of CNTs caused the retardation of PEF’s carbonization process. This led to a slightly longer lifetime under isothermal conditions at higher temperatures, while at ambient temperature the PEF nanocomposites’ lifetime is shorter, compared to neat PEF.

## 1. Introduction

Sustainability has become a crucial concept for both scientists and industries considering the increasing greenhouse gas (GHG) emissions. The production and combustion of petroleum-derived polymers hold a significant share in the total GHG emissions [1,2]; furthermore, recent studies have shown a concerning increase of microplastics in the marine environment [3,4] while they have already integrated into some widely consumed food products [5]. In this context, biobased polymers have been developed to replace conventional petroleum-derived plastics in several applications. Therefore, building blocks produced from renewable feedstocks such as succinic acid (SA) [6], vanillin [7], L-lactic acid (LLA) [8], 2,5-furandicarboxylic acid (FDCA) [9], etc., have been the center of scientific attention for the production of eco-friendly, non-toxic plastics.

More specifically, FDCA has attracted the most interest as a replacement for terephthalic acid in oil-based polyesters due to their structural resemblance [10]. Thus, FDCA-based polyesters such as Polyethylene 2,5-furandicarboxylate (PEF) [11,12], Poly(propylene 2,5-furandicarboxylate) (PPF) [13,14,15], Poly(butylene 2,5-furan dicarboxylate) (PBF) [15], as well as co-polyesters such as Poly(1,4-butylene-co-1,4-cyclohexanedimethylene-2,5-furandicarboxylic acid) (PBCFs) [16], have been extensively studied in terms of their physicochemical properties. Of the above-biobased polyesters, PEF is currently the one that has been commercially produced [17], due to its superior mechanical and gas barrier properties compared to its petroleum-based polyethylene terephthalate (PET) [11,18,19]. 

Despite PEF’s exceptional characteristics, such as high glass transition temperature, low melting temperature, gas barrier, and mechanical properties compared to PET, it is characterized by a slow crystallization rate [20], which limits its processing and application fields. Optimizing a desired physical property of a polymer can be achieved via copolymerization or the introduction of an appropriate filler in the polymer matrix. In the latter case, several studies have been published that focus on the thermal and mechanical properties of furan-based polyester composites. More specifically, PPF, PBF, and PEF/clays nanocomposites have been synthesized in the past, and it has been shown that the introduction of small amounts of nanoclays imposes a heterogenous nucleation effect and accelerates the overall crystallization process of the polymer matrix, together with enhancing its thermal stability [21,22,23]. 

Carbon nanofillers have also been used as reinforcing fillers in furan-based polyester matrices. Graphene nanoplatelets (GNPs) and fumed silica nanoparticles (SiO_2_) were inserted in low amounts, in Poly(hexylene 2,5 furan-dicarboxylate) (PHF) matrix individually and combined, forming hybrid nanocomposites, and it was found that both fillers accelerated the crystallization rate and crystalline fraction of PHF [24]. PEF nanocomposites reinforced with neat and functionalized Multi-Walled Carbon Nanotubes (MWCNTs) and with Graphene Oxide (GO) were also in situ prepared; all the nanocomposites presented increased nucleation density, due to the heterogeneous nucleation of each filler. The X-ray Diffraction (XRD) measurements revealed that in the solvent-treated neat PEF, β-crystals were formed, while the nanocomposites presented peaks were associated with the α-crystal phase [25]. PPF/GNPs nanocomposites’ thermal degradation study revealed that GNPs incorporation in the polymer matrix resulted in slightly higher thermal degradation activation energy, while at the same time, more prominent homolytic degradation reactions occur in the case of the nanocomposites compared to neat PPF [26]. 

Although there has been considerable research on the thermal behavior of nanocomposites of furan-based polyesters in the past few years, the fillers’ effects on the structure of the polymer matrix have not been thoroughly studied. Furthermore, almost no research regarding the lifetime of PEF nanocomposites in an ambient atmosphere is known to the knowledge of the authors. The lifetime of a material is a crucial property that directly affects its service life while also being an indication of a faster or slower decomposition when disposed of in the environment. To this end, PEF nanocomposites containing GNPs, CNTs, and hybrids comprising both fillers were in situ synthesized. Previous studies of the prepared PEF/GNPs and PEF/CNTs nanocomposites indicated the high nucleation efficiency of both fillers and their reinforcing effect on the matrix’s nanomechanical properties [27,28,29]. Therefore, to obtain a more complete picture of each filler’s effect on the physical properties of PEF, the matrix’s crystallographic parameters, the effect of the ultrasonication process on each filler state, and the polymer’s lifetime when GNPs, CNTs, and both fillers are incorporated into the biobased polyester were examined in this work. To evaluate the structural characteristics of neat PEF and structural changes induced in its nanocomposites by the presence of each filler, X-ray Diffraction (XRD), Fourier transform infrared spectroscopy–attenuated total reflectance (FTIR–ATR), and X-Ray Photoelectron Spectroscopy (XPS) were employed, while Transmission Electron Microscopy (TEM) was used to assess the morphology and structural properties of a hybrid PEF nanocomposite, as well. Furthermore, the thermo-oxidative behavior of neat PEF and selected PEF nanocomposites was studied by means of Thermogravimetric Analysis (TGA). Lifetime predictions of the selected samples were performed using the Activation Energy (Ε_α_) values of the thermo-oxidative degradation derived from the isoconversional method of Friedman, at ambient atmosphere. As previously mentioned, lifetime predictions are a useful tool for the estimation of the material’s lifetime performance. 

## 2. Materials and Methods

### 2.1. Materials 

Multi-walled carbon nanotubes were used as a reinforcing filler for the synthesis of PEF/CNTs and PEF/GNPs/CNTs nanocomposites. The multi-walled CNTs are synthesized by chemical vapor deposition (CVD) and have a nominal diameter of 30−80 nm, a length of about 10 μm, and a specific surface area of around 60 m^2^/g with a carbon content >99.9%. The filler was supplied by Timesnano Chengdu Organic Chemicals Co. (China). Graphene nanoplatelets (GNPs) GNPs under the trade name xGNP M5 with an average thickness of 6 to 8 nm and an average diameter of 5 μm were supplied by XG Sciences Inc. (Lansing, Michigan, USA). The average surface area varies from 120 to 150 m^2^/g; the bulk density of the GNPs is 2200 kg/m^3^.

### 2.2. Synthesis of Poly(ethylene 2,5-furandicarboxylate) and Its Nanocomposites

PEF’s crystal density has been reported to be 1.548  ±  008 g/cm^3^, while the amorphous one is equal to 1.434  ±  003 g/cm^3^ [30]. The thermal and mechanical properties of the prepared materials in this study have been reported in previous publications [28,29]. PEF/GNPs, PEF/CNTs, and hybrid PEF/GNPs/CNTs nanocomposites have been prepared in this work by the two-stage transesterification/polycondensation method in a glass batch reactor as described in our previous work [27]. Before polymerization, ethylene glycol (EG) and the appropriate amount of the selected filler (as listed in Table 1) were sonicated for 20 min (50 Watt, 30 kHZ, cycle 1, amplitude 100%-intermediate time breaks were made to avoid excessive heating) (Hielscher UP50H) to create a uniform dispersion. The nanocomposites are referred to in this manuscript, as seen in Table 1. 

### 2.3. Characterization Methods

Intrinsic viscosity [η] measurements were conducted to calculate the average molecular weight of the prepared PEF materials out of three measurements, as described in our previous work [27].

### 2.4. X-rays Diffraction Measurements

The XRD patterns of PEF nanocomposites powder samples, prepared by mechanical grinding, were obtained at Bragg–Brentano geometry using a water-cooled Rigaku Ultima_+_ (company: Rigaku Corporation, Tokyo, Japan) diffractometer with CuK_a_ radiation and a step size of 0.05° step with a delay time of 3 s, operating at 40 kV and 30 mA. Experimental error is approximately equal to the step size (±0.05°). Prior to the measurements, all the initially amorphous materials were annealed at 423 K for 3 h. In order to obtain the necessary parameters derived from the XRD patterns of each material, peak profile analysis was conducted prior to the calculations described above. All XRD patterns were initially baseline-corrected. Subsequently, selected peaks of each material were fitted using Gaussian-Lorentzian Cross Product. The fitting parameters were then collected and used for further analysis. The peak profile of the diffractograms was conducted using the PeakFit software (SigmaPlot Co., Slough, UK). 

### 2.5. Fourier Transform Infrared Spectroscopy—Attenuated Total Reflectance (FTIR–ATR) Measurements 

The FTIR spectra of the annealed (at 423 K) PEF samples were collected with a Cary 670 spectroscope from Agilent Technologies (Palo Alto, Santa Clara, CA, USA) equipped with a diamond attenuated total reflectance (ATR) accessory (GladiATR, Pike Technologies, Madison, WI, USA). The measurements were conducted in the mid-IR area (4000–400 cm^−1^), with 16 scans and 4 cm^−1^ resolution. The experimental error of the measurements is approximately equal to the resolution (±4 cm^−1^). These results have been presented in our previous work [27] and will be mentioned in the present manuscript.

### 2.6. X-ray Photoelectron Spectroscopy (XPS)

XPS spectra of the selected PEF samples were collected with an Axis UltraDLD system by Kratos Analytical using an Al-Ka1 X-ray source (energy 1486.6 eV) with pass energy on the analyzer of 160 eV for survey scans and 20 eV for high-resolution spectra. The spectra shifting, due to surface charging effects, was fixed, and the binding energies were calibrated using the C-C bonds from the C 1 s orbital at 284.6 eV (±0.2 eV). The spectra decomposition was conducted using a Gauss/Lorentz product function and a Shirley function to subtract the signal background, with the least-squares fitting method. Atomic ratios were calculated from background-subtracted peak areas using the relative sensitivity factors provided by the data analysis system (Vision 2.2.10, developed by Kratos analytical, Manchester, UK).

### 2.7. Transmission Electron Microscopy (TEM)

The morphological and structural properties of a hybrid PEF nanocomposite sample were investigated by Transmission Electron Microscopy (TEM) methods using a Jeol JEM 1010 electron microscope operated at 100 kV. TEM specimens were prepared by sectioning the samples in a Leica UCT Ultracut ultramicrotome and collecting thin sections on 400-mesh Cu grids.

### 2.8. Thermogravimetric Analysis (TGA)

The thermo-oxidative behavior of selected PEF samples was studied by Thermogravimetric Analysis of all the selected PEF samples using the SETARAM SETSYS TG-DTA 16/18 setup by heating the samples under 50 mL/min of air flow rate. Kinetic Analysis was conducted for the selected PEF samples; the materials were heated at four different heating rates, 5, 10, 15, and 20 K/min, according to the ICTAC recommendations [31], under a dry air environment. Samples were placed in alumina crucibles, and an empty alumina crucible was used as a reference. Continuous recordings of sample temperature, sample weight, and its first derivative were collected. 

## 3. Results

The average molecular weight of a polymer plays a crucial role in its final thermal, mechanical, and structural properties. Since the synthesis method of the prepared PEF materials was in situ polymerization in laboratory equipment, the resulting average molecular weights may vary. Consequently, the average molecular weights of neat PEF and PEF/carbon fillers nanocomposites were calculated by intrinsic viscosity, as described in our previous work [27]. The molecular weights of PEF/GNPs, PEF/CNTs PEF/CNTs, and hybrid PEF/0.25 GNPs/CNTs nanocomposites are presented in Table 2. It must be noted that the M_n_ values of the prepared polyesters may vary depending on the polymerization process, i.e., solid-state polymerization following the two-stage transesterification/polycondensation has been reported to increase the PEF’s molecular weight depending on the catalyst and the selected experimental conditions [32]. Ring-opening polymerization of PEF also resulted in high molecular weight polyesters up to approximately 79,000 g/mol [33]. Previous studies regarding PEF nanocomposites synthesized via the two-stage transesterification/polycondensation method have reported M_n_ values between 7700–22,000 [25,34,35]. Therefore, the resulting molecular weights in this work are within the above range.

### 3.1. Structural Characteristics of Neat PEF and PEF Nanocomposites

Incorporating carbon fillers in a polymer can induce changes in the crystalline and molecular structure of the matrix, and therefore, a structural analysis of the prepared PEF materials has been conducted to obtain further information. For this reason, XRD, FTIR-ATR, and XPS measurements were employed to investigate the effect of GNPs, CNTs, and their synergistic effect on the crystalline phase and molecular conformation of the PEF matrix. 

**Figure 1:**
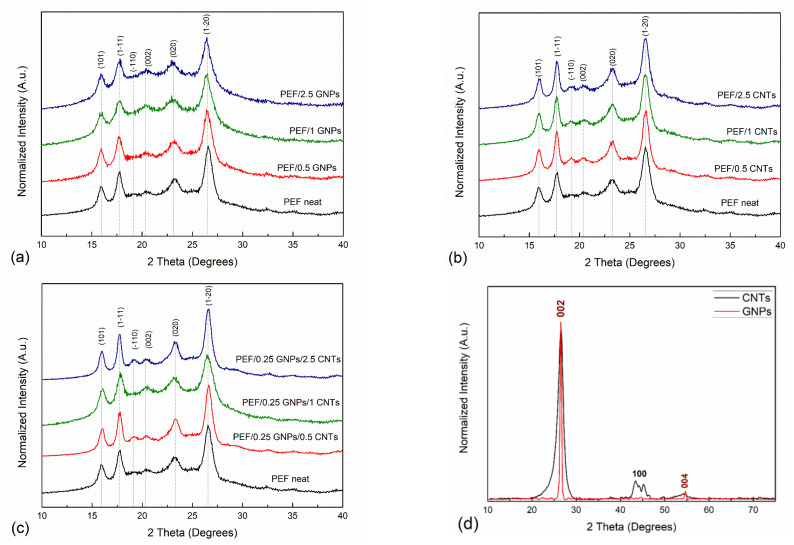
XRD patterns of neat PEF with (**a**) PEF/GNPs, (**b**) PEF/CNTs, and (**c**) PEF/0.25 GNPs/CNTs nanocomposites, and (**d**) the diffractogram of the incorporated GNPs and CNTs.

The XRD patterns of the pristine GNPs and CNTs that were incorporated in the PEF matrix are presented in Figure 1. The main diffraction peaks of GNPs appear at 26.55° and 54.65° correspond to (002) [(0002)] and (004) [(0004)] planes of the graphitic structure with a d-spacing value of 3.36 Å and 1.68 Å, respectively, which agrees with the reported literature values [36,37]. The corresponding CNTs diffractogram presents diffraction peaks at 26.45°, 43.45°, and 54°, corresponding to the (002) [(0002)], (100) [(10-10)], and (004) [(0004)] crystallographic planes, respectively. The (002) and (004) planes correspond to a d-spacing of 3.35 Å and 1.675 Å, respectively, while the (100), which originated from the two-dimensional lattice of the CNTs, corresponds to a d-spacing of 2.08 Å, which is in agreement with the reported literature values [38]. It can be observed that the diffraction peaks of CNTs are wider compared to GNPs, suggesting the formation of smaller crystals [38], as will be shown from the TEM images later in the manuscript. 

Figure 1a–c present the XRD patterns of cold crystallized at 418 K neat PEF with PEF/GNPs, PEF/GNPs, PEF/CNTs, and PEF/0.25 GNPs/CNTs nanocomposites, respectively. All the PEF samples present six diffraction peaks at 2θ: 15.9°, 17.7°, 19.2°, 20.4°, 23.2°, and 26.6°, corresponding to (101), (1-11), (-110), (002), (020), and (1-20) of the triclinic α-phase of PEF, correspondingly [39]. At first glance, it can be observed that the crystallographic peaks of PEF/CNTs and PEF/0.25 GNPs/CNTs nanocomposites appear to be slightly sharper compared to neat PEF and PEF/GNPs nanocomposites, suggesting finer crystal formation in these cases. To further evaluate the crystal structure of all the prepared PEF materials, the unit cell parameters, the lamellar thickness oriented along the reported crystallographic planes, and the crystallinity degree were calculated.

The interplanar spacings of a material’s diffraction peaks were calculated from the Bragg diffraction formula:

(1) n · λ=2 · d · sinθ

where *n* indicates the reflection order, *λ* is the wavelength of CuK_α_ radiation (*λ* = 1.5406 Å) [40], and *θ* is the diffraction peak’s angle. When the crystal system of a material is known, the unit cell constants, namely the a, b, and c unit cell vectors, then α, β, and γ angles can be obtained. The obtained values were calculated in this work using the Jade software (Materials Data Inc., Livermore, CA, USA).

Peak profile analysis was applied in this work to calculate the crystal size (*L_hkl_*) using Scherrer’s equation [41] (Equation (2)):

(2) $$B_{2\theta }=\frac{K\lambda }{L_{hkl}cos\theta }$$

where *B* is the full-width half maximum of the corresponding peak at angle *θ* and *K* is the geometrical factor, here equal to 0.9 [42]. The crystal size calculations were applied for the main peaks of all the prepared materials (neat PEF and its nanocomposites). 

The crystallinity degree of a semicrystalline material can be calculated from the XRD patterns. Amorphous content in a sample is identified by the presence of a broad halo in the XRD pattern. The weight crystalline fractions of all the prepared annealed materials were calculated in this work using the equation proposed by Matthews et al. [43]:

(3) $$X_{c}=\frac{A_{cr}}{A_{cr}+A_{am}}\;\cdot \;100\%$$

where *X_c_* is the crystallinity degree and *A_cr_* and *A_am_* correspond to the calculated areas of crystalline and amorphous peaks, respectively.

Table 3 presents the lattice parameters of neat PEF and all the prepared PEF nanocomposites. Several studies focus on the structural characteristics of PEF. In 1968, Kazaryan et al. firstly proposed the triclinic cell with dimensions of a = 5.75 Å, b = 5.35 Å, c = 20.1 Å and α = 113.5°, β = 90°, and γ = 112° [44]. However, later, these proposed unit cell parameters have been dismissed due to causing a false positioning of the main peaks in the XRD pattern [39]. A more recent study by Mao et al. proposed, for the case of PEF fibers, a monoclinic cell with unit cell parameters of a = 5.784 Å, b = 6.780 Å, c = 20.296 Å, and γ = 103.3° [45]. Recently, however, Maini et al. investigated the polymorphs of PEF obtained by usual methods, such as the one used in this work [39]. They have concluded that in the case of PEF’s α-phase the triclinic cell is proposed with the unit cell parameters of a = 5.729 Å, b = 7.89 Å, c = 9.62 Å, α = 98.1°, β = 65.1°, γ = 101.3° [39]. Considering the above, the unit cell parameters a, b, and c, and their mutual angles α, β, and γ were calculated, and are close in value to the ones reported by Maini et al. The a, b, and c unit cell lengths values do not present significant deviations between neat PEF and PEF nanocomposites in any case of incorporated carbon filler; the same applies to the calculated β and γ angles. On the other hand, the α angle, which is the angle between the b and c axis, presents higher values in the case of the PEF nanocomposites (1–2° increase) when compared to neat PEF. This augmentation does not seem to be related to each filler’s content. The altered values of α angle suggest that incorporating any of the selected carbon fillers imposes a minor slide between the molecular segments that form the unit cell along the c-axis. The unit cell volume of all the prepared PEF materials was also calculated. No significant variations of V were observed in the case of PEF/GNPs and PEF/CNTs nanocomposites compared to the neat PEF. However, hybrid PEF/0.25 GNPs/CNTs unit cell volume slightly increases with increasing CNTs content compared to the rest of the PEF samples. 

It has been reported that the lamellar thickness of a polymer increases with molecular weight [46,47]. To investigate the effect of both molecular weight and each filler’s content on the crystal size of the PEF matrix, the lamellar thickness of the polymer crystals oriented across (101), (1-11), (-110), (002) (020), and (1-20) planes were calculated by the Scherrer–Debye equation and are presented in Table 3. For the sake of accuracy and minimization of experimental errors, the lamellar thickness corresponding to the most distinct diffraction peaks, i.e., (101), (1-11), and (1-20), will be taken into account to analyze the crystal behavior of the PEF nanocomposites. The calculated crystal thicknesses of the prepared PEF samples do not seem to follow a specific trend related to their average molecular weight. Therefore, it is assumed that the fillers’ effect is the predominant factor that controls the lamellar thicknesses’ behavior in the PEF nanocomposites. 

The crystallization process is controlled by two factors: nucleation, where heterogeneous nucleation is favored in the case of an incorporating filler, and diffusion, where the macromolecular chains migrate to the surface of a formed nucleus. Introducing a filler particle can affect both factors, either by facilitating the crystal growth or constraining it. Regarding the PEF nanocomposites, the values of the calculated lamellar thicknesses of PEF/GNPs nanocomposites decrease with increasing filler content. Similar findings have been reported in the case of HDPE/GNPs nanocomposites [48]. This behavior is attributed to the hindrance of the molecular chains’ diffusion to the surface of the existing nuclei caused by the GNPs, despite the heterogeneous nucleation effect of the platelets on the polymer chains.

On the other hand, the PEF/CNTs nanocomposites present a rather different behavior; the lamellar thicknesses are higher than neat PEF and increase with increasing the CNTs content. Similar behavior has been observed in the case of iPP/CNTs nanocomposites [49,50]. The two-dimensional character of the CNTs facilitates the initial heterogeneous nucleation of the PEF molecular chains and does not obscure the migration of the polymer chains to the surface of the formed nuclei, thus allowing further crystal growth. In the case of the hybrid PEF nanocomposites, the GNPs and CNTs appear to have a competitive effect on the lamellar thicknesses of PEF. Compared to neat PEF, all hybrid nanocomposites present thicker lamellae. However, these values are lower than their PEF/CNTs nanocomposites counterparts for 0.5 and 1 wt.% of CNTs. The small number of GNPs (0.25 wt.%) is enough to constrain the crystal growth of the PEF matrix; nonetheless, this effect is diminished when the CNTs content of the hybrid nanocomposites reaches 2.5 wt.%.

**Figure 2:**
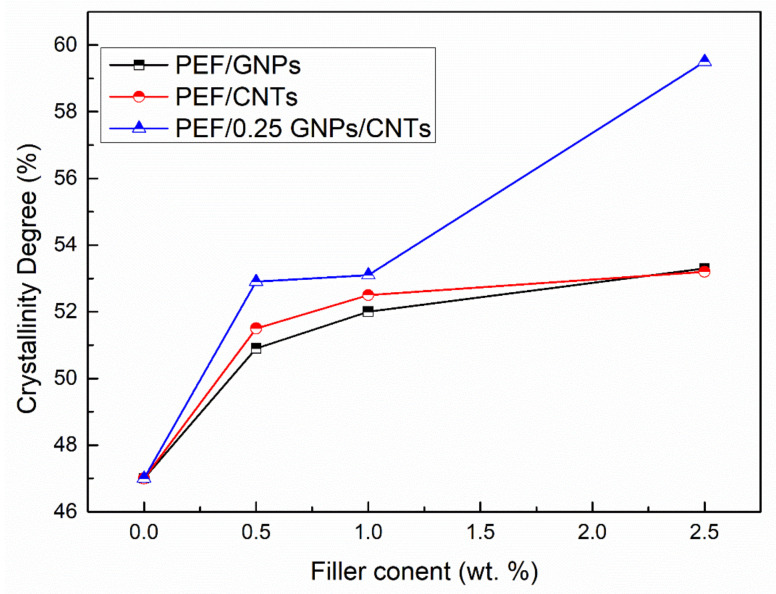
Crystallinity degree vs. filler content of PEF/GNPs, PEF/CNTs, and PEF/0.25 GNPs/CNTs nanocomposites.

The crystallinity degree of the annealed PEF materials has been calculated using Equation (3), and the results are reported in Table 4 and illustrated in Figure 2. In any case of the PEF nanocomposites, the X_c_ values increase with the augmentation of the filler. PEF/CNTs present slightly higher crystalline fraction values than the PEF/GNPs counterparts, with the exception of 2.5 wt.% filler content, which presents the same value. Increased crystallinity in polymers results in improved mechanical properties; in our previous works, the crystalline PEF/GNPs and PEF/CNTs nanocomposites presented significantly higher hardness and elastic modulus values when compared to their amorphous counterpart and neat PEF [28,29]. This behavior is attributed to the highly ordered lamellae which are characterized by a more significant intermolecular bonding leading to an enhanced hardness, strength, and elastic modulus. The hybrid nanocomposites show significantly higher crystallinity degree values at any CNTs content compared to the PEF/CNTs and PEF/GNPs nanocomposites counterparts, suggesting that the heterogeneous nucleation was promoted due to the larger number of nucleating sites, i.e., each filler’s particles, and/or due to the contribution of the crystalline peaks of the fillers which are not clearly visible in the respective diffractograms; this will be mentioned later in the manuscript.

**Figure 3:**
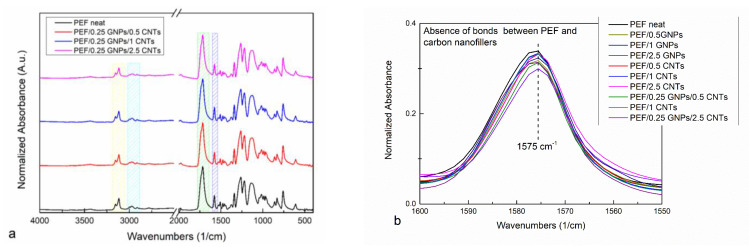
ATR spectra of neat PEF with (**a**) PEF/0.25 GNPs/CNTs and (**b**) enlarged ATR spectra of the ester bond band of all the PEF materials.

ATR measurements were also conducted on the annealed (at 423 K) prepared PEF materials. It has been reported that the crystalline and amorphous regions of PEF are sensitive to specific conformational preferences observed through their absorbance spectrum [51]. A possible bond formation between the incorporated fillers and the PEF matrix can also be confirmed or disproved by the ATR spectra of the PEF nanocomposites. The ATR absorbance spectra of neat PEF with PEF/GNPs and PEF/CNTs have been presented in our previous work [27], and the corresponding ones of PEF/0.25 GNPs/CNTs nanocomposites are presented in Figure 3a. No noticeable differences have been observed between the neat PEF and PEF/Carbon nanofillers nanocomposites. The main absorbance peaks around 3245–3045 cm^−1^ and 3045–2865 cm^−1^ (noted with yellow and Turquoise squares, respectively) are attributed to the C-H asymmetric and symmetric stretching of the furan ring’s carbon–hydrogen bonds, and the methylene group’s asymmetric stretching of the EG fragment of the PEF’s molecule. The large peak between 1840–1615 cm^−1^ corresponds to the stretching of C=O ester bonds, while the peak at 1575 cm^−1^ is assigned to the asymmetric stretching of the C=C double bond on the furan ring. The latter bond is reported to be sensitive to the polymer–filler interactions [52], i.e., when bonds between the incorporated filler and the polymer matrix are formed, the peak migrates to lower wavenumbers. As seen in Figure 3b, no migration of the aforementioned peak has been observed, and therefore, it can be suggested that no chemical interactions between the incorporated filler and the PEF matrix are present, as has been already mentioned in our previous work for the PEF/GNPs and PEF/CNTs nanocomposites [27]. 

Additional information regarding the crystalline and amorphous state of the PEF materials can be derived through the preferred polymer chain’s conformation, which can be observed at the ATR spectra. More particularly, it has been reported by Araujo et al. that the preferred conformations of the FDCA and EG fragments for the semicrystalline and amorphous PEF are the syn^FDCA^-trans^EG^ and anti^FDCA^-gauche^EG^ (helix) conformations, respectively [51]. The trans conformation of the EG fragment (O−CH_2_−CH_2_−O) is characterized by a dihedral angle of 180° and the gauche conformation by a dihedral angle of 60° [51]. Accordingly, the syn and anti-isomerisms of PEF’s molecule FDCA fragment (O_ring_−C_ring_−C=O) have a dihedral angle of 0° and 180° [51]. 

Figure S1a,b present the ATR spectra of the prepared PEF materials at 1500–1320 cm^−1^ and 680–540 cm^−1^ wavenumber range. The absorbance peak of 1340 cm^−1^ results from the wagging vibrations of CH_2_ groups in trans-EG units [51]. The gauche EG segments, which are more prominent in the amorphous PEF, cause the shift of the later vibration to ≈1375 cm^−1^. Accordingly, the deformation of trans-CH_2_ groups presents an absorbance band at 1477 cm^−1^, while the corresponding one of the gauche conformation shifts to ≈1455 cm^−1^ [51]. As seen in Figure S1a, both conformations are visible on both neat and nanocomposites PEF materials, confirming the semicrystalline character of the annealed samples. The FDCA fragment’s anti and syn isomerisms of PEF yield a broad absorbance band between 630 and 580 cm^−1^ as a result of the ring deformation mode, presented in Figure S1b [51]. The syn isomerism of the crystalline region is represented by the major peak at 609 cm^−1^, while the shoulder peak around 621 cm^−1^ is assigned to the FDCA fragment’s anti-isomerism of amorphous regions [51]. An enlarged image of the later peak of all the prepared PEF materials is presented in Figure S1b(ii); the bold dotted line represents the neat PEF spectrum region. It is observed that the shoulder peak of 621 cm^−1^ is more distinct compared to the ones of PEF/Carbon nanofillers, suggesting that the anti-isomerism found in the amorphous region of PEF is in greater amount, i.e., the nanocomposites are characterized by a higher degree of the crystalline fraction compared to neat PEF, as previously concluded by the XRD analysis. 

**Figure 4:**
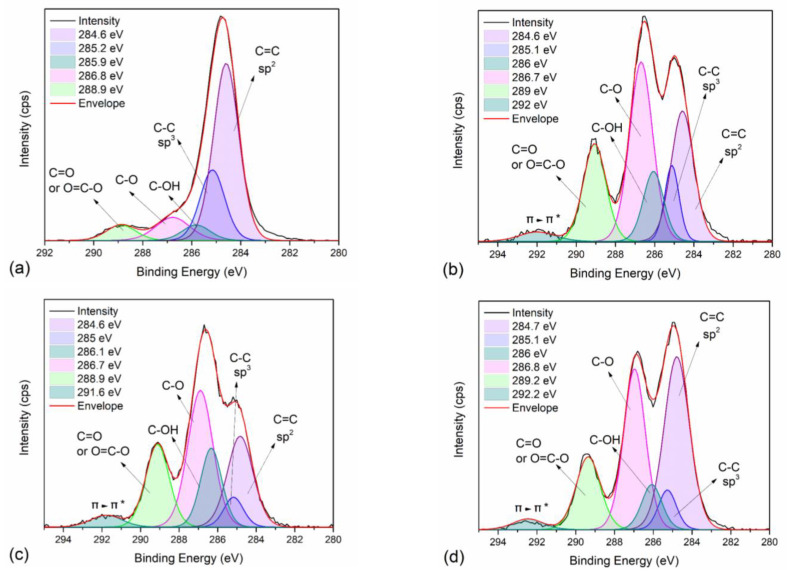
XPS spectra and peak deconvolution centered on the C1s orbital range of (**a**) neat PEF (R^2^ = 0.997), (**b**) PEF/1 GNPs (R^2^ = 0.996), (**c**) PEF/1 CNTs (R^2^ = 0.998), and (**d**) PEF/0.25 GNPs/1 CNTs (R^2^ = 0.997).

To further study the intermolecular interactions of neat PEF and PEF/Carbon nanofillers, XPS measurements were employed on selected PEF samples. Figure S2a–d present the wide scan of neat PEF, PEF/1 GNPs, PEF/1 CNTs, and PEF/0.25 GNPs/1 CNTs, respectively, while the corresponding atomic concentration of carbon and oxygen are reported in Table S1. Small amounts of Si were found on the surface of the understudied samples, possibly due to glass contamination from the glass flask. Oxygen’s atomic concentration of neat PEF is lower than those of the PEF nanocomposites. It is known that hydroxyl, epoxy, and carbonyl groups are attached to the graphitic sheets of GNPs and CNTs to a small degree. However, the ratio of carbon to oxygen for the PEF nanocomposite is considerably lower compared to the one of neat PEF. The C1s and O1s peaks deconvolution of all the selected materials was conducted to examine the later behavior of the nanocomposites further. Figure 4a–d present the C1s and Figure S3a–d present the O1s peak deconvolution of neat PEF, PEF/1 GNPs, PEF/1 CNTs, and PEF/0.25 GNPs/1 CNTs, respectively, while the corresponding binding energies and areas of each deconvoluted peak are reported in Table 5 and Table 6, for the C1s and O1s core levels. The deconvoluted carbon peaks found in all the PEF samples at binding energies of 284.6, 285.2, 286, 286.8, and 289 eV (±0.2 eV error values) are attributed to the C=C sp^2^ and C-C sp^3^ carbon hybridization, and the C-OH, C-O, and C=O/O=C-O bonds [53,54], respectively. In the case of the PEF nanocomposites, an extra peak is found at 292 eV attributed to the π-π* transition of the GNPs’ and CNTs’ graphitic layers [55]. Accordingly, the deconvoluted oxygen peaks were found near 531.4, 532, and 533.6 eV for all the PEF materials and correspond to the O-**C=O**, C-OH, and **O-C**=O bonds, respectively [53,54]. The sp^2^ to sp^3^ ratio for neat PEF is close to 3, confirming the 6 C-C bonds with sp^2^ and 2 C-C bonds with sp^3^ hybridization on the PEF monomer; incorporating any of the carbon nanofillers, the corresponding ratio increases due to the sp^2^ carbon hybridization of the graphitic layers of the GNPs and CNTs. 

To a minor degree, the increase in oxygen atomic concentration and the carbon–oxygen bonds on the XPS spectra of the nanocomposites is attributed to the addition of the carbon nanofillers, as earlier suggested. Since the ATR spectra of the PEF nanocomposites did not suggest the formation of a chemical bond between the filler and the polymer matrix, this possibility is dismissed. 

Prior to the polymerization process of PEF nanocomposites, the selected fillers’ dispersion was achieved by their ultrasonication in ethylene glycol. Dispersion of a nanofiller succeeded due to the propagation of the sound waves in altering high and low-pressure cycles through the particles’ aggregates. More specifically, in the case of GNPs and CNTs, during the low-pressure cycles, vapor bubbles are generated in the EG, increasing in size (cavitations). These generated cavitations consecutively collapse throughout the high-pressure cycles releasing high mechanical and thermal energy which causes a temperature increase in the EG and causes the splitting of the larger particles/aggregates. Insertion of the EG molecules between the graphene layers also takes place, causing the GNPs exfoliation [56]. EG molecules can also be embedded in the CNTs cavities during the ultrasonication process. 

As earlier stated, pause-time was applied during ultrasonication of the EG/GNPs or/and CNTs dispersion to avoid excessive heating and the increase of the fluid’s viscosity. During the process, the collapse of the microcavities causes a local increase in pressure and temperature. Given that both of the nanofillers used are characterized by high thermal conductivity, the temperature throughout the fluid’s volume increases rapidly. Pause time was also introduced to avoid viscosity augmentation, especially in the case of hybrid nanocomposites. It has been shown that by increasing the filler’s loading in the liquid medium and applying ultrasonication, the viscosity increases depending on the sonication time [57]; above a time threshold, the viscosity decreases, due to the finer particles formed during the process. Long-time exposure to ultrasound was avoided in this work, to avoid the excessive formation of structural and topological defects on the GNPs and CNTs (amorphous sp^3^-carbon formation, excessive exfoliation, unzipping of the nanotubes to graphenoids, length reduction) [58]. Therefore, the introduced time breaks in the case of the PEF/GNPs and PEF/CNTs were 30 sec per 5 min, while in the case of the PEF/0.25 GNPs/CNTs they were raised to 1 min per 3 min. 

Ultrasonication can also induce oxygenated species, aside from defects, onto the graphene sheets, as suggested by Skaltsas et al. [59]. The presence of carboxylic acids and ether oxides introduced on the surface of the exfoliated graphene sheets or CNTs was found to be independent of the liquid medium. Thus, the increase in oxygen atomic concentration and the carbon–oxygen bonds on the XPS spectra could be attributed to the introduction of the functionalities on each filler’s surface. The longer pause period that was applied during the ultrasonication process (1 min instead of 30 sec) in the case of the hybrid nanocomposites, as well as the shorter application time (3 instead of 5 min), is possibly the cause of the smaller oxygen atomic concentration compared to the PEF/1 GNPs and PEF/1 CNTs nanocomposites. During ultrasonication, the shorter cycle time of application does not allow the same degree of the nanoparticles’ dispersion or functionalization, as in the rest of the nanocomposites. Additionally, the longer pause period might cause several neighboring dispersed nanoparticles (GNPs or CNTs) to re-agglomerate due to van der Waals’s attractions. Consequently, the following cycle of sonication causes the breaking of these new aggregates, possibly lowering the degree of further functionalization of each filler’s particles. 

**Figure 5:**
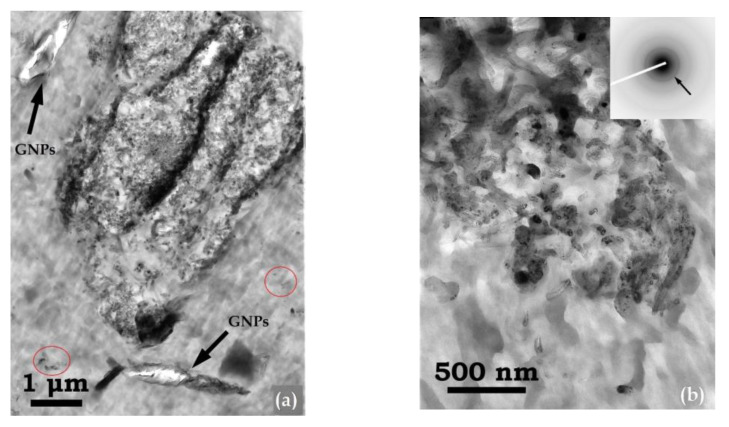

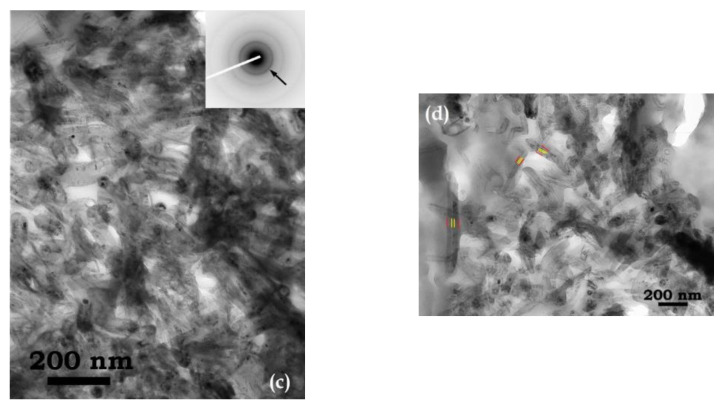
(**a**) TEM image of the PEF/0.25 GNPs/1 CNTs hybrid nanocomposite showing a large CNT aggregate, along with neighboring GNPs. In (**b**,**c**), the corresponding SAED ring patterns of CNT bundles are shown as insets, whereby the (0002) graphitic planes are denoted by arrows. In (**d**), the outer and inner diameters of individual CNTs are depicted by red and yellow lines, respectively.

TEM imaging of the hybrid nanocomposite PEF/0.25 GNPs/1 CNTs was implemented to investigate the CNTs’ morphological and structural characteristics when GNPs are also present in the PEF matrix. In Figure 5, TEM micrographs of the hybrid PEF nanocomposite are shown, where both carbon fillers seem to be effectively embedded in the PEF matrix (Figure 5a). The majority of CNTs are organized in the form of large aggregates, with diameters in the range of 200 nm to several μm, comprising large numbers of CNTs that appear bowed and twisted, forming a bundle-like structure. The corresponding ring-type selected area electron diffraction (SAED) patterns of the observed bundles (insets in Figure 5b,c) confirm that they consist of randomly oriented crystalline CNTs. Here, the (0002) graphitic planes of the CNTs are indicated by arrows. Moreover, the continuous and rather broad form of the rings in the SAED patterns suggests that the dimensions of crystalline CNTs are in the nanoscale range (<100 nm) [60]. Indeed, as seen in Figure 5d, measurements of the nanotubes’ average outer (red lines) and inner (yellow lines) diameters resulted in values of 57.7 ± 5.2 nm and 9.16 ± 1.4 nm, respectively. Despite the formation of large CNT aggregates, it was observed that these bundles were homogeneously distributed throughout the PEF matrix. Furthermore, GNPs, consisting of a few single crystalline graphene layers, were also observed in the vicinity of the CNT bundles (Figure 5a), regardless of their very low density. These observations comply with our initial assumption, i.e., incorporating both fillers in the matrix may result in large aggregates, due to the different ultrasonic procedures followed to avoid excessive heating and increased viscosity (see above).

### 3.2. Thero-Oxidative Behavior of Neat PEF and PEF Nanocomposites

**Figure 6:**
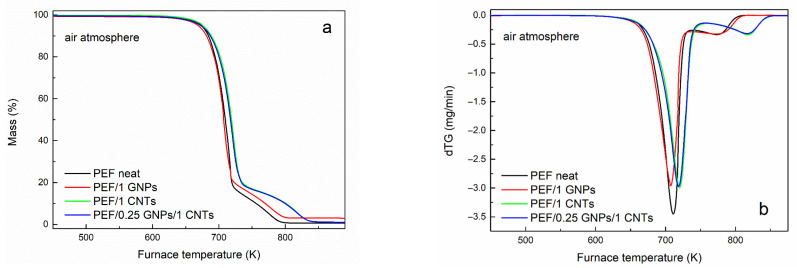

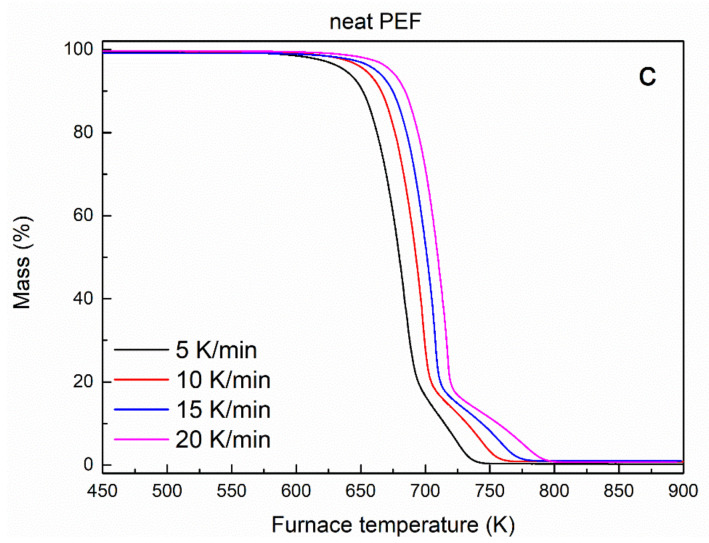
Mass loss curves of (**a**) neat PEF, PEF/1 GNPs, PEF/1 CNTs, and PEF/0.25 GNPs/1 CNTs nanocomposites and (**b**) its first derivative, versus temperature at a heating rate of 20 K/min, and (**c**) neat PEF at 5, 10, 15, and 20 K/min heating rates versus temperature under dry air atmosphere.

The thermo-oxidative degradation behavior of neat PEF, PEF/1 GNPs, PEF/1 CNTs, and PEF/0.25 GNPs/1 CNTs was also evaluated by conducting TGA measurements under a controlled dry air atmosphere. Figure 6a,b present the mass loss and dTG curves of the understudy PEF materials versus temperature, respectively. The temperatures at 2% and 50% mass loss and the peak temperatures of the dTG curve of neat PEF and its nanocomposites are presented in Table 7. As seen from the dTG curves, the thermo-oxidative degradation takes place in two steps, where the first occurs between 500–730/750 K and is related to the random scission of the ester linkage, and the second one (730/750–870 K) occurs with a subsequent reaction of carbonization [61]. As seen in Table 7, the temperature at which the thermo-oxidative degradation begins (T_2%_) does not present large deviations between the understudy PEF materials. PEF/1 CNTs nanocomposite appears to be the most thermally stable, while PEF/1 GNPs degrade earlier in temperature compared to the rest of the PEF samples. In terms of thermal stability, the first step’s temperatures that the maximum degradation rate for PEF/1 CNTs and PEF/0.25 GNPs/1 CNTs rate occur are at slightly higher temperatures compared to neat PEF and PEG/1 GNPs; at the same time, the dTG peak of the second degradation step seems to be affected to a greater degree by the presence of the CNTs compared to the first peak. PEF/1 CNTs and PEF/0.25 GNPs/1 CNTs nanocomposites present the second dTG peak at considerably higher temperatures than neat PEF and PEF/GNPs.

The exact reactions and mechanism of PEF’s thermo-oxidative degradation have not been studied in detail yet and are expected to be complex. However, the most probable scenario is that the thermo-oxidative degradation of PEF might be similar to the one of PET, i.e., the initial reaction with oxygen at elevated temperatures involves oxidation at the α-methylene carbon resulting in the formation of hydroperoxide, and homolytic cleavage of the oxygen–carbon bond adjacent to the ester, which results in the formation of aliphatic and benzoic (in the case of PET) acids [62]. 

The thermo-oxidative degradation’s effective activation energy of neat PEF, PEF/1 GNPs, PEF/1 CNTs, and PEF/0.25 GNPs/1 CNTs was calculated by the isoconversional differential method of Friedman. Generally, isoconversional analysis can be applied without any assumption on the reaction mechanism and, for this reason, is referred to as a model-free method. 

**Figure 7:**
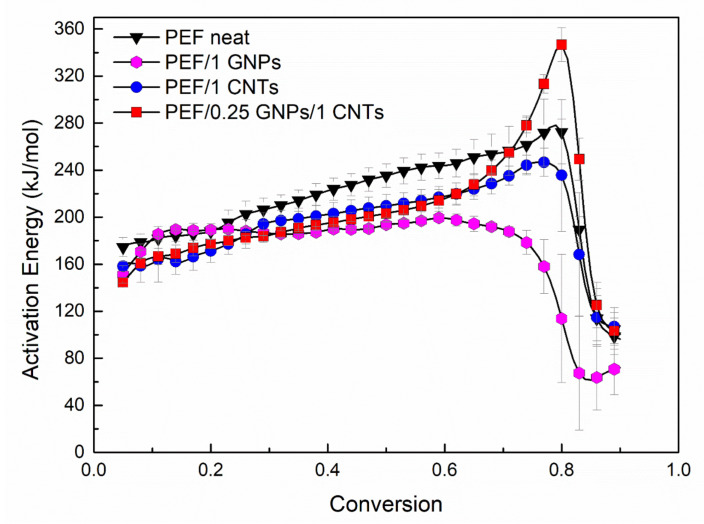
Thermal degradation’s effective activation energy of neat PEF, PEF/1 GNPs, PEF/1 CNTs, and PEF/0.25 GNPs/1 CNTs versus degree of conversion under dry air atmosphere.

Isoconversional methods follow the isoconversional principle, which declares that the reaction rate is a function of temperature at a constant conversion α, as follows [31]:

(4) $${\left[\frac{dln\left(\frac{da}{dt}\right)}{dT^{-1}}\right]}_{a}=-\frac{E_{a}}{R}$$

where *α* stands for the values related at a given extent of conversion of the process and is calculated by the following equation [63]:

(5) $$a=\frac{m_{0}-m_{t}}{m_{0}-m_{f}}$$

where *m*_0_ and *m_f_* are the sample’s initial and final weight, and *m_t_* is the sample’s weight at time (*t*). According to the isoconversional methods, the activation energy remains constant with temperature and varies with varying degree of conversion [64]. The isoconversional methods are categorized into differential and integral methods. The most commonly used differential method is the one proposed by Friedman and is given by the following equation [31]:

(6) $$ln{\left(\frac{d\alpha }{dt}\right)}_{\alpha }=constant-\frac{\Delta E_{\alpha }}{R\cdot T_{\alpha }}$$

where *α* stands for the values related at a given extent of conversion. By plotting *ln*(*dα/dt*) versus 1/*T_α_*, the value of −Δ*E_α_/R* for a given degree of crystallinity can be obtained by the slope of the fitted line. The activation energy values of the selected PEF samples versus the degree of conversion are presented in Figure 7.

For neat PEF, the values of E_α_ increase with the degree of conversion almost linearly until α = 0.8 and then abruptly shift to lower values (≈100 kJ/mol). Similar behavior is observed in the case of PEF/1 CNTs, while for PEF/1 GNPs, the activation energy is almost constant until α < 0.7 and then drops to 60 kJ/mol. On the contrary, PEF/0.25 GNPs/1 CNTs present rather different behavior. In the initial and middle stages of the degradation, increasing the degree of conversion, the activation energy increases as in the case of neat PEF and PEF/1 CNTs until α = 0.7. When the extent of conversion is between 0.7 and 0.8, during the second step of the thermo-oxidative degradation, the Ε_α_ values increase rapidly, and for α > 0.8 they drop to 120 kJ/mol. These changes in the Ε_α_ values at the later stages of degradation (α > 0.6) in any case of the PEF materials correspond to the second step of the degradation, the carbonization reaction. Therefore, in the case of the hybrid PEF nanocomposite, the presence of small amounts of GNPs together with CNTs creates a synergistic effect and increases the energy barrier for the carbonization step. The lowest activation energy for the later stages of the degradation is observed for the PEF/1 GNPs nanocomposite, while the highest is for PEF/0.25 GNPs/1 CNTs, as earlier stated. The initial and middle stages of the degradation do not deviate significantly regarding the activation energy values between the neat PEF and nanocomposites. 

### 3.3. Lifetime Prediction

Kinetic Analysis can be used to estimate the lifetime of a material. The lifetime of a material is defined as the time after which a material’s property decays to such an extent that it cannot fulfill the desired application’s function. Using TGA, one can predict the lifetime of a sample when it is exposed to heat and deteriorates due to thermal aging. However, the aging of a material can be caused or accelerated by other factors such as humidity, pressure, mechanical stress, etc. Therefore, the mass loss measurements of TGA can provide limited information on the decay of other properties, such as the mechanical behavior of a polymer. Nevertheless, it is known that a polymer’s mechanical properties deteriorate when the macromolecular chains start to break before the formation of low molecular mass volatile products causes a detectable mass loss in TGA. Thus, one can express a limiting decay of a property in terms of a specific extent of conversion value as an assumption, i.e., when α = 0.05, the deterioration of physical properties is considered significant, thus the lifetime of a material can be approximated by calculating the time needed by a material under a temperature T to reach this value of the conversion [31]. 

Lifetime predictions can take place using experiments under any temperature program. Depending on the temperature program, one can select a different approach. Model-based and model-free methods have been proposed to estimate the lifetime of a material. In this work, the resulting E_α_ derived by the isoconversional method of Friedman was used to estimate the lifetime of selected PEF materials under continuous air flow. The predictive equation was proposed as follows [65]:

(7) $$t_{a}=\frac{\int_{0}^{T_{a}}\exp\left(-\frac{E_{a}}{RT}\right)dT}{\beta \exp\left(-\frac{E_{a}}{RT_{0}}\right)}$$

where *t_α_* is the predicted lifetime, *T_α_* is the temperature at which the selected *α* is reached, and *T*_0_ is the selected isothermal temperature at which the prediction is calculated. Due to the lack of a model’s assumption, the method is called model-free prediction. Its main advantage is that it applies to processes for which the effective activation energy varies with the degree of conversion *α*, thus providing more reliable results [31]. 

Thus, using the results from the isoconversional analysis for the thermo-oxidative degradation of neat PEF, PEF/1 GNPs, PEF/1 CNTs, and PEF/0.25 GNPs/1 CNTs, lifetime predictions were conducted using Equation (7). Through this analysis, the time needed for the understudy material to reach a selected thermo-oxidative degradation’s extent of conversion under isothermal conditions can be calculated. The selected degree of conversions was set to α = 0.05 (approximately 5% mass loss), which corresponds to the early stages of thermo-oxidative decomposition [66,67], and the materials’ properties (mechanical, thermal) begin to deteriorate. Therefore, one can calculate a product’s lifetime at the operational temperature, depending on the application, and evaluate the overall endurance of the material through time. 

**Figure 8:**
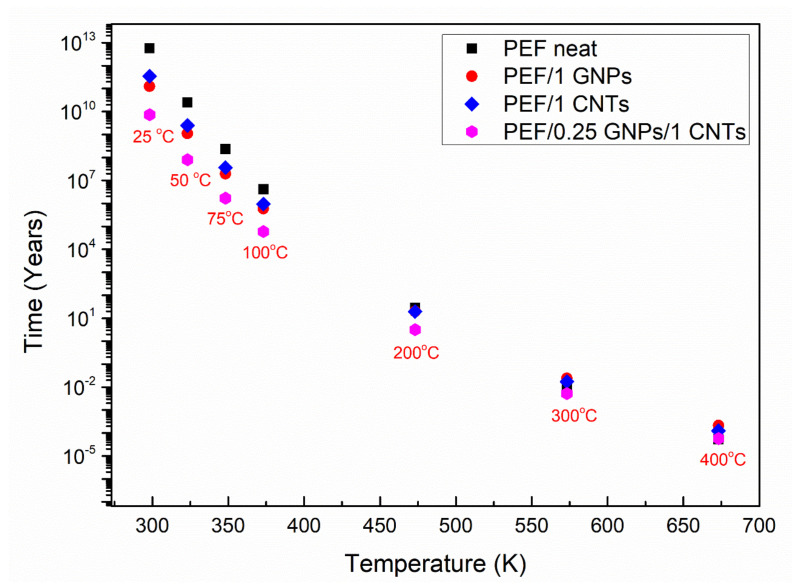
Lifetime predictions of neat PEF, PEF/1 GNPs, PEF/1 CNTs, and PEF/0.25 GNPs/1 CNTs nanocomposites for various selected temperatures under a dry air environment.

Figure 8 presents the lifetime in years versus selected temperatures for neat PEF, PEF/1 GNPs, PEF/1 CNTs, and PEF/0.25 GNPs/1 CNTs. As seen, the lifetime decreases with increasing temperature for all the understudy materials, as expected. When the temperature is 298 K (25 °C), neat PEF’s thermo-oxidative degradation begins after 5.8 × 10^12^ years, while the corresponding lifetimes for PEF/1 GNPs, PEF/1 CNTs, and PEF/0.25 GNPs/1 CNTs are 1.3 × 10^11^_,_ 3.5 × 10^11^, and 7.3 × 10^9^ years, respectively. Therefore, considering that the corresponding effective activation energy values for neat PEF, PEF/1 GNPs, PEF/1 CNTs, and PEF/0.25 GNPs/1 CNTs, when α = 0.05 are 175, 151, 158, and 145 kJ/mol, it can be seen that the lifetime for the given conversion follows the same trend. The above results indicate that the nanocomposite PEF materials deteriorate faster when exposed to ambient temperatures, i.e., when the materials are disposed of to the environment as plastic waste which is a desired trait. As the temperature increases, the lifetime of the understudy PEF materials presents smaller deviations from one another, while there is a turning point temperature at 573 K, where PEF/1 GNPs and PEF/1 CNTs present a higher lifetime than neat PEF. More specifically, neat PEF presents a lifetime equal to ≈4.5 days, while the corresponding lifetimes for PEF/1 GNPs, PEF/1 CNTs, and PEF/0.25 GNPs/1 CNTs nanocomposites are ≈8.8, 6.3, and 1.9 days, respectively. Finally, when the temperature reaches 673 K, the corresponding lifetimes for neat PEF, PEF/1 GNPs, PEF/1 CNTs, and PEF/0.25 GNPs/1 CNTs are 27.9, 113.6, 65.7, and 30.4 min, meaning that with increasing temperature, the lifetime of the nanocomposites exceeds the one of neat PEF, while at lower temperatures, neat PEF is more stable. However, it must be noted that these estimations are made based on the assumption that the temperature is always stable, and the environment consists only of dry air under a constant flow rate without taking into consideration other parameters such as the light (UV radiation), humidity, possible microorganisms’ presence, mechanical loadings, etc. [67]. When the other parameters are present, the lifetime of each material reduces significantly, but the above analysis is a satisfactory first approach to the service life predictions of the prepared PEF materials. 

## 4. Conclusions

The study of GNPs and CNTs’ effect on the structural characteristics and thermo-oxidative degradation on the PEF matrix was conducted in this work. More specifically, a series of PEF/GNPs, PEF/CNTs, and hybrid PEF/0.25 GNPs/CNTs were in situ prepared by the two-stage transesterification/polycondensation method. X-ray diffraction measurements of the prepared materials showed that the introduction of each filler resulted in a higher crystalline fraction compared to neat PEF, which contributes to the reinforcement of PEF’s mechanical properties, while the incorporation of CNTs resulted in higher lamellar thicknesses with increasing the CNTs content. It was also found that, in the case of the nanocomposites, the α angle presents higher values, suggesting that the fillers impose a minor slide between the molecular segments that form the unit cell along the c-axis. XPS spectra of selected PEF nanocomposites indicate the formation of a larger amount of carbon–oxygen bonds compared to neat PEF due to the functionalization of the carbon nanofillers which took place during the ultrasonication process. TEM observations of PEF/0.25 GNPs/1CNTs nanocomposite revealed that incorporating both fillers in the matrix resulted in large CNTs aggregates, possibly due to the different ultrasonic procedures followed in the case of the hybrid nanocomposites. The thermo-oxidative degradation of neat PEF and selected PEF nanocomposites was studied using TGA. The incorporation of CNTs in the PEF matrix mainly affected the second step of the thermo-oxidative degradation, which corresponds to the reaction of carbonization. The effective activation energy (E_α_) of the process for the selected samples was calculated using the isoconversional method of Friedman. Using the calculated E_α_ values, the lifetime of each sample under selected temperature conditions and an atmosphere of air was estimated. It was shown that at ambient temperatures, the lifetime of neat PEF is higher than the one of the nanocomposites, meaning that when the prepared PEF nanocomposites decompose faster when disposed of to the environment as waste, there is a turning point temperature at 573 K, where the nanocomposites present a higher lifetime than neat PEF, suggesting a higher endurance under high temperatures. The above findings are useful to enlighten the filler’s effect on a polymer matrix in terms of its structure and thermo-oxidative behavior, and the way the dispersion procedure can affect the state of the filler in the polymer matrix. Considering the above along with the findings of our previous work, the reinforced mechanical properties of the PEF nanocomposites, along with their higher crystallization rate, make the prepared materials suitable for applications in the automobile industry. Future work on their electrical properties could reveal that these PEF nanocomposites may also be used in applications such as flexible electronics. 

## Figures and Tables

**Table 1:** PEF nanocomposites fillers’ content and their given names in this work.

PEF Nanocomposite Series	GNPs x Content (wt.%)	CNTs y Content (wt.%)
PEF/x GNPs	0.5	0
	1	
	2.5	
PEF/y CNTs	0	0.5
		1
		2.5
PEF/x GNPs/y CNTs	0.25	0.5
		1
		2.5

**Table 2:** Average molecular weight values of neat PEF and PEF/carbon fillers nanocomposites.

Sample	${\bar{M}}_{n}\;(g/\mathrm{mol})$
neat PEF	12,000 ± 78
PEF/0.5 GNPs	8000 ± 23
PEF/1 GNPs	10,600 ± 24
PEF/2.5 GNPs	8000 ± 16
PEF/0.5 CNTs	8300 ± 34
PEF/1 CNTs	14,600 ± 12
PEF/2.5 CNTs	8000 ± 23
PEF/0.25 GNPs/0.5 CNTs	9100 ± 24
PEF/0.25 GNPs/1 CNTs	10,700 ± 12
PEF/0.25 GNPs/2.5 CNTs	14,500 ± 35

**Table 3:** Calculated unit cell parameters of neat PEF and PEF/Carbon fillers nanocomposites. The estimated standard deviation is written in parentheses.

Sample	a (Å)	b (Å)	c (Å)	α (°)	β (°)	γ (°)	V (Å^3^)
neat PEF	5.744(3)	7.785(2)	9.759(5)	94.38(3)	63.33(2)	101.42(4)	382.24
PEF/0.5 GNPs	5.763(5)	7.805(6)	9.667(4)	95.58(2)	64.3(4)	101.43(3)	383.96
PEF/1 GNPs	5.756(2)	7.783(3)	9.747(4)	96.18(2)	63.97(4)	101.93(4)	383.77
PEF/2.5 GNPs	5.76(3)	7.779(4)	9.75(2)	95.66(4)	63.38(2)	101.86(2)	382.21
PEF/0.5 CNTs	5.752(4)	7.78(4)	9.625(3)	96.53(2)	64.43(5)	101.78(2)	381.7
PEF/1 CNTs	5.752(2)	7.792(3)	9.671(2)	95.86(6)	63.77(2)	101.96(3)	381.33
PEF/2.5 CNTs	5.75(3)	7.807(5)	9.71(3)	96.19(3)	64.1(3)	101.7(2)	383.85
PEF/0.25 GNPs/0.5 CNTs	5.735(5)	7.791(4)	9.733(5)	96.19(2)	64.43(3)	101.74(5)	384
PEF/0.25 GNPs/1 CNTs	5.73(4)	7.814(2)	9.683(2)	96.08(4)	64.81(2)	101.62(3)	384.14
PEF/0.25 GNPs/2.5 CNTs	5.754(6)	7.813(3)	9.778(3)	96.82(2)	64.86(4)	101.52(2)	389.6

**Table 4:** The calculated lamellar thickness of the main crystallographic planes and crystallinity degree of neat PEF and PEF/carbon fillers nanocomposites, along with the R^2^ values of the fittings.

Sample	L_(101)_ (Å)	L_(1-11)_ (Å)	L_(-110)_ (Å)	L_(002)_ (Å)	L_(020)_ (Å)	L_(1-20)_ (Å)	X_c_ (%)	R^2^
neat PEF	19.24	20.73	12.2	18.11	14.9	16	47	0.995
PEF/0.5 GNPs	16.3	17.58	4.01	5.96	8.87	13.05	50.9	0.996
PEF/1 GNPs	13.58	14.32	7.36	9.05	6.58	11.37	52	0.996
PEF/2.5 GNPs	12.71	13.62	10.6	9.67	6.52	11.05	53.3	0.995
PEF/0.5 CNTs	23.89	27.77	16.8	17.21	15.74	17.85	51.5	0.997
PEF/1 CNTs	21.32	24.36	14.2	17	14.15	17.4	52.5	0.995
PEF/2.5 CNTs	22.35	25.72	16	22.85	15.92	17.62	53.2	0.995
PEF/0.25 GNPs/0.5 CNTs	19.52	23.54	14.2	18.59	15.92	17.85	52.9	0.997
PEF/0.25 GNPs/1 CNTs	19.52	23.15	15	19.36	15.74	18.08	53.1	0.997
PEF/0.25 GNPs/2.5 CNTs	23.49	27.78	19.6	19.92	14.44	17.85	59.5	0.996

**Table 5:** Binding energies and % area of the carbon bonds at the C1s spectra of neat PEF, PEF/1 GNPs, PEF/1 CNTs, and PEF/0.25 GNPs/1 CNTs.

Sample	C=C sp^2^		C=C sp^3^		C-OH		C-O		C=O		π→π*	
	BE (eV)	Area (%)	BE (eV)	Area (%)	BE (eV)	Area (%)	BE (eV)	Area (%)	BE (eV)	Area (%)	BE (eV)	Area (%)
PEF neat	284.6	56.9	285.2	22	285.9	5.4	286.8	9.9	288.9	5.8	-	-
PEF/1 GNPs	284.6	24.4	285.1	8.9	286	11.7	286.7	34	289	18.2	292	2.8
PEF/1 CNTs	284.6	22.6	285	5.6	286.1	16.1	286.7	33.2	288.9	18.8	291.6	3.6
PEF/0.25 GNPs/1 CNTs	284.7	37.7	285.1	6.1	286	7.8	286.8	30.3	289.2	15.3	292.2	2.8

**Table 6:** Binding energies and % area of the oxygen bonds at the O1s spectra of neat PEF, PEF/1 GNPs, PEF/1 CNTs, and PEF/0.25 GNPs/1 CNTs.

Sample	C=O		C-OH		C-O	
	BE (eV)	Area (%)	BE (eV)	Area (%)	BE (eV)	Area (%)
PEF neat	531.3	61.4	532.1	12.2	533.6	26.4
PEF/1 GNPs	531.5	30.4	532.2	14.8	533.6	54.7
PEF/1 CNTs	531.4	21.1	532	18.7	533.6	60.2
PEF/0.25 GNPs/1 CNTs	531.6	31.7	532	11.1	533.6	57.2

**Table 7:** Temperatures at 2% and 50% mass loss and the peak temperatures of the dTG curve of neat PEF and its nanocomposites under a dry air atmosphere.

Sample	T_2%_ (K)	T_50%_ (K)	T_dTG1_ (K)	T_dTG2_ (K)	Residual Mass (%)
neat PEF	652.9	709.2	711	773	0.7
PEF/1 GNPs	642.2	707.1	707.3	772.5	3
PEF/1 CNTs	655.2	719	720	817.8	0.93
PEF/0.25 GNPs/1 CNTs	646.4	717.3	718.6	816.6	1.2

## Data Availability

The data presented in this study are available on request from the corresponding author.

## Supplementary Materials

The following supporting information can be downloaded at: https://www.mdpi.com/article/10.3390/polym15020401/s1, Figure S1: ATR spectra of all the prepared PEF materials (a) at the area of the EG absorbance bands and (b) the FDCA absorbance bands; Figure S2: XPS wide scan spectra of (a) neat PEF, (b) PEF/1 GNPs, (c) PEF/1 CNTs, and (d) PEF/0.25 GNPs/1 CNTs; Table S1: The atomic concentration of carbon and oxygen on the surface of neat PEF, PEF/1 GNPs, PEF/1 CNTs, and PEF/0.25 GNPs/1 CNTs; Figure S3: XPS spectra and peak deconvolution, centered on the O1s orbital range of (a) neat PEF, (b) PEF/1 GNPs, (c) PEF/1 CNTs, and (d) PEF/0.25 GNPs/1 CNTs.
