# Supplementary material for: Incorporating Graphene Nanoplatelets and Carbon Nanotubes in Biobased Poly(ethylene 2,5-furandicarboxylate): Fillers’ Effect on the Matrix’s Structure and Lifetime
Source: Polymers (Basel). 2023 Jan 12;15(2):401. doi: 10.3390/polym15020401 (PMC9863989; doi:10.3390/polym15020401)
Supplement: Supplementary file 1 [file polymers-15-00401-s001.zip › polymers-2107669-supplementary.pdf]

Supplementary Materials

Incorporating Graphene Nanoplatelets and Carbon Nanotubes in Biobased Poly(ethylene 2,5-furandicarboxylate): Fillers' Effect on the Matrix's Structure and Lifetime

Dimitra Kourtidou [1], Dimitrios Karfaridis [1], Thomas Kehagias [1], George Vourlias [1], Dimitrios N. Bikiaris [2,*] and Konstantinos Chrissafis [1,*]

[1] Laboratory of Advanced Materials and Devices, School of Physics, Aristotle University of Thessaloniki, Thessaloniki GR-541 24, Greece

[2] Laboratory of Polymer Chemistry and Technology, Department of Chemistry, Aristotle University of Thessaloniki; Thessaloniki GR-541 24, Greece

* Correspondence: dbic@chem.auth.gr (D.N.B.); hrisafis@physics.auth.gr (K.C.)

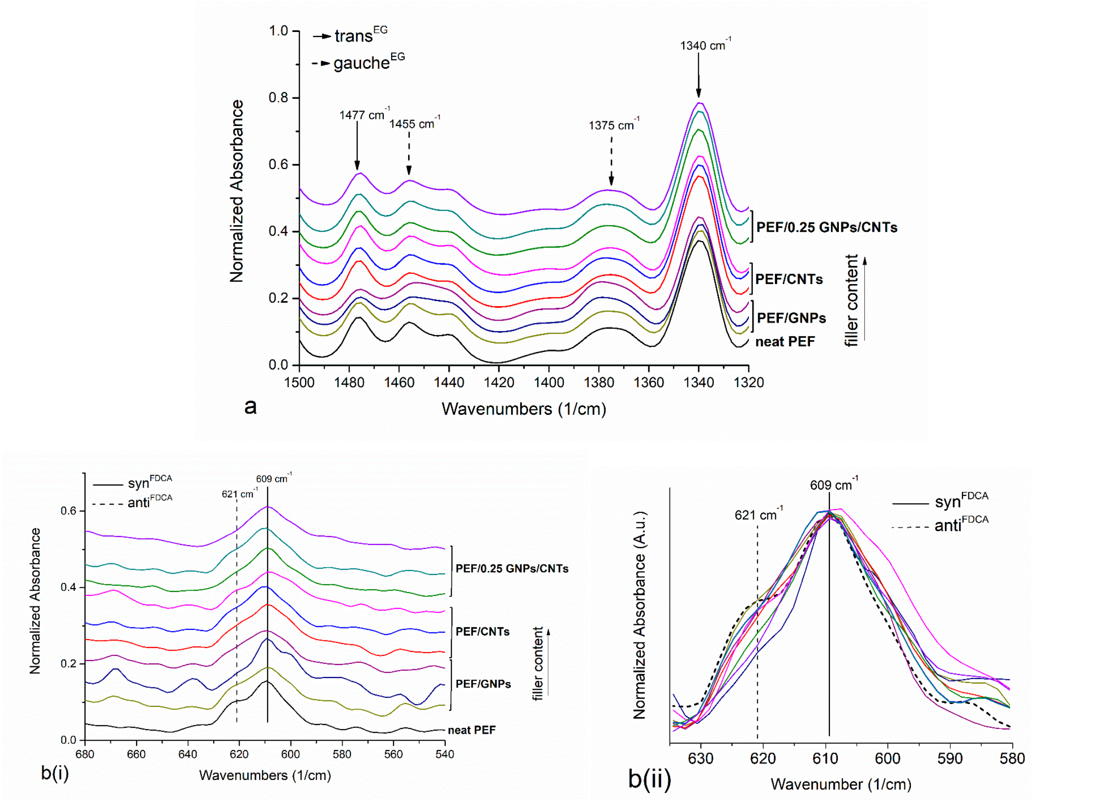

Figure S1. ATR spectra of all the prepared PEF materials (**a**) at the area of the EG absorbance bands and (**b**) the FDCA absorbance bands.

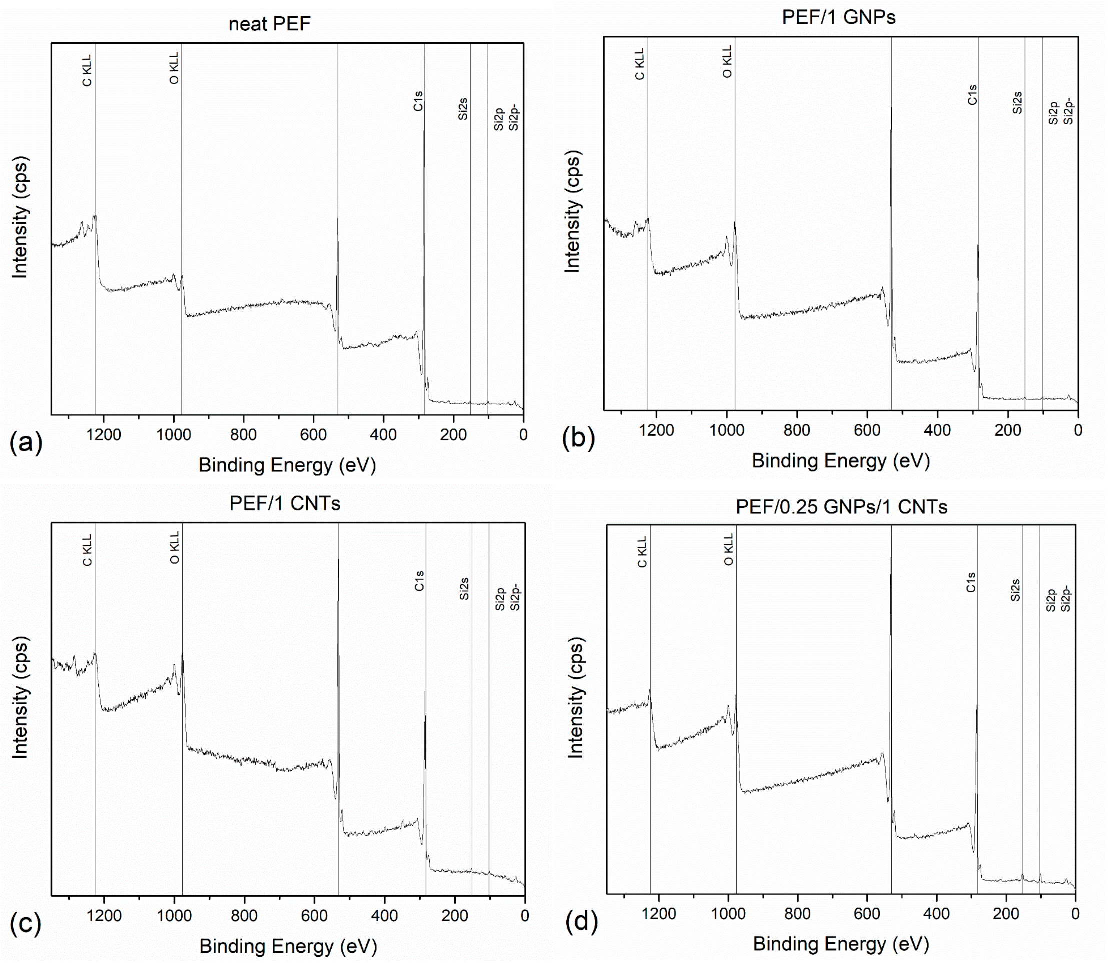

Figure S2. XPS wide scan spectra of (**a**) neat PEF, (**b**) PEF/ 1 GNPs, (**c**) PEF/1 CNTs, and (**d**) PEF/0.25 GNPs/1 CNTs.

Table S1. The atomic concentration of carbon and oxygen on the surface of neat PEF, PEF/ 1 GNPs, PEF/1 CNTs, and PEF/0.25 GNPs/1 CNTs

Sample	C atomic conc. (%)	O atomic conc. (%)
PEF neat	85.4	14.6
PEF/1 GNPs	66.1	33.9
PEF/1 CNTs	64.7	35.2
PEF/0.25 GNPs/1 CNTs	72.3	27.7

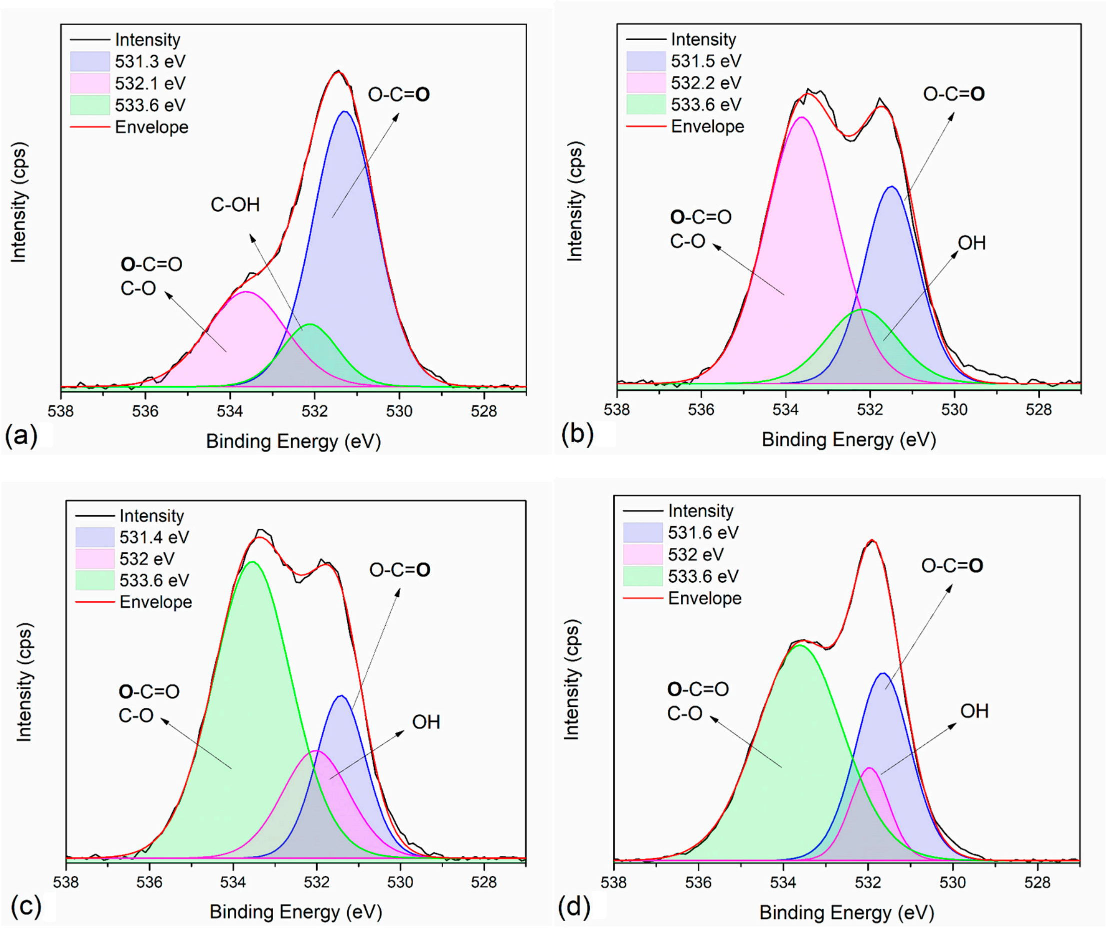

Figure S3. XPS spectra and peak deconvolution centered on the O1s orbital range of (**a**) neat PEF (R^2=0.999), (**b**) PEF/ 1 GNPs (R^2=0.996), (**c**) PEF/1 CNTs (R^2=0.997), and (**d**) PEF/0.25 GNPs/1 CNTs (R^2=0.997).
